# Development of Goal Management Training^+^ for Methamphetamine Use Disorder Through Collaborative Design

**DOI:** 10.3389/fpsyt.2022.876018

**Published:** 2022-04-25

**Authors:** Alexandra C. Anderson, Alex H. Robinson, Eden Potter, Bronte Kerley, Daphne Flynn, Dan I. Lubman, Antonio Verdejo-García

**Affiliations:** ^1^School of Psychological Sciences, Turner Institute for Brain and Mental Health, Monash University, Melbourne, VIC, Australia; ^2^Monash Addiction Research Centre, Monash University, Melbourne, VIC, Australia; ^3^Design Health Collab, Monash Art, Design and Architecture, Monash University, Melbourne, VIC, Australia; ^4^Turning Point, Eastern Health Clinical School, Monash University, Melbourne, VIC, Australia

**Keywords:** Goal Management Training, cognitive remediation, user engagement, person-based, participatory design, collaboration, addiction, methamphetamine

## Abstract

**Background:**

Methamphetamine use disorder (MUD) is associated with executive dysfunctions, which are linked with poorer treatment outcomes including earlier drop out and relapse. However, current treatments for MUD do not address executive functions. Goal Management Training (GMT) is an evidence-based cognitive remediation program for executive dysfunction, although required modifications to enhance its relevance and application within addiction treatment settings. This study aimed to (1) tailor GMT to the key cognitive deficits and typical treatment duration of MUD; (2) explore consumers' (people with MUD) engagement with the revised program; (3) implement a prototype of the program with consumers; and (4) present the manualized standard administration to clinical service providers.

**Methods:**

We followed the Medical Research Council Complex Interventions Framework and employed an evidence- and person-based intervention development process. We used a four-phased approach and collaborated with neuropsychology experts, design researchers in healthcare, consumers with MUD, and clinical service providers. Each aim was addressed in a separate study phase; including content refinement and review with neuropsychology experts (phase 1), intervention design and collaboration with consumers (phase 2), prototype development and review with consumers (phase 3), and final program modifications and review with clinical stakeholders (phase 4).

**Results:**

Findings from phase 1 indicated support for targeting four cognitive processes (attention, impulse control, goal setting, and decision-making). Key feedback included the need to help habitualize cognitive strategies and to guide consumers in applying these strategies in emotionally salient situations. Findings from phases 2 and 3 indicated consumer support for the program strategies and materials but highlighted the need to further enhance the personal relevance of specific content and journal activities. Findings from phase 4 provided clinicians support for the revised program but indicated an opportunity to minimize unintended effects. We present the intervention materials for the final revised program, Goal Management Training^+^ (GMT^+^), in line with TIDieR guidelines.

**Conclusions:**

GMT^+^ targets key cognitive processes and is sensitive to the clinical needs of people with MUD. Our intervention development process was important for informing the active ingredients and materials for GMT^+^, and indicated initial consumer and provider acceptability prior to conducting a clinical trial.

## Introduction

Methamphetamine is a highly addictive stimulant that presents a global public health concern (1). In 2019, ~27 million people had used amphetamines worldwide, and there is growing concern around the rise of harmful patterns of use (2). Methamphetamine use disorder (MUD) is associated with greater risk of suffering physical and mental health conditions, including cardiovascular disease, blood-borne viruses, psychosis, depression, and suicide, as well as social disadvantage (3–5). Underlying the hallmark characteristics of MUD (i.e., loss of control over drug intake, escalation of use despite growing negative consequences) are cognitive deficits in executive functions (the higher-order cognitive skills that orchestrate goal-directed behaviors) (6, 7).

Emerging research has revealed that executive functions, such as inhibitory control, working memory and decision-making, are significantly associated with MUD treatment outcomes (8, 9). Specifically, consumers with deficits in executive functions are at greater risk of dropping out of treatment, relapsing after abstinence-oriented treatment programs, and struggling to regain quality of life (10, 11). This research, together with recent evidence showing that current treatment interventions for MUD have overall limited efficacy (4), raises the need to incorporate cognitive remediation interventions for executive dysfunction as an add-on to current treatment approaches (12, 13). Cognitive remediation interventions aim to strengthen executive functions via meta-cognitive skills and strategy learning within a therapeutic group environment (14).

In a recent meta-analysis of cognitive-boosting interventions for addiction treatment, we showed that Goal Management Training (GMT) is the most promising approach to ameliorate executive deficits in this context (15). GMT was originally developed to improve executive functions in brain injury populations (16), but its active ingredients, such as strategies to prevent disinhibited responses and manage complex tasks, are well-suited for substance use disorders (17–20). However, the original GMT presents three key limitations in the context of MUD treatment. Firstly, the length of the program is 7–9 weeks, which almost doubles the standard duration of treatment episodes for MUD (21). Second, the training activities and their delivery were not designed to address the nature and severity of cognitive deficits in substance use disorders or MUD specifically. In MUD, deficits are less pronounced than in brain injury, and there is a need for a greater emphasis on aiding long-term decision-making and inhibiting impulsive behaviors, which are key predictors of addiction treatment outcomes (8). Third, the presentation of materials (including character examples, design, and activities) may lack engagement potential for people with MUD. For example, the original program was designed to suit older adults with different demographics and may not be adequate to capture attention or personal relevance for people with MUD. In addressing these limitations, it is important to incorporate the views of people who use methamphetamine to enhance the intervention experience for the end-consumers (22) and to ultimately increase the chance of it being considered as “helpful” and acceptable.

The purpose of this study was to develop a modified version of Goal Management Training (now Goal Management Training^+^; GMT^+^) to strengthen executive function and improve clinical outcomes in individuals with MUD. Our specific aims were:

**Aim 1**: To develop an updated version of GMT tailored to the cognitive deficits of people with MUD and the duration of typical treatment episodes for MUD/substance use disorders.
**Aim 2**: To gather consumers' (i.e., people with MUD) engagement with the updated GMT program.
**Aim 3**: To implement a consumer-acceptable prototype of the program (i.e., GMT^+^).
**Aim 4:** To manualize the intervention, showcase a standard administration among clinicians, and prepare materials for the proof-of-concept pilot trial.

## Materials and Methods

### Design and General Procedures

We followed the Medical Research Council Complex Interventions Framework (23). The intervention development process was underpinned by an evidence- and person-based approach. This approach aims to ground the development of interventions in a deep understanding of the consumer perspective, with consideration to their psychosocial context (24). Importantly, the process involves a flexible approach that is guided by understanding the needs, goals, and desires of the end consumers (i.e., people with MUD) (25). This is achieved through involving consumers in the development process as experts of their own life experiences, within a participatory design framework (26). The intervention development process incorporates multidisciplinary skills and perspectives by including cognitive and clinical psychology researchers (core research team), neuropsychology researchers, design researchers in healthcare, consumers with MUD, and clinical service providers.

We used a qualitative approach and developed the intervention in four phases. Phase 1 involved content refinement, where the existing content was assessed for relevance for MUD and reorganized in a streamlined set of modules/sessions to bring it closer to the standard duration of MUD treatment. We then conducted a focus group with experts in neuropsychology to evaluate these changes and seek further recommendations. This was considered the intervention planning phase. Phase 2 involved intervention design. We collaborated with design researchers in healthcare to reimagine the materials and visual identity for GMT^+^, as well as to increase the experiential engagement of activities involving the core GMT skills. Next, we conducted a focus group with consumers with MUD to gain feedback on the design and engagement with the key concepts and activities. Intervention development took place over phases 3 and 4. Phase 3 involved developing a prototype of the intervention. We conducted a second focus group with people with MUD via teleconferencing software, to review the changes from the first focus group and to test sample activities from the module and daily journal. Phase 4 included the final program modifications, including feedback gained in a review session with clinicians. We present a description of the final materials, in line with TIDieR guidelines (27).

The Monash University Human Research Ethics Committee (12364) and Eastern Health Human Research Ethics Committee (LR19/023) approved the study and all participants provided informed consent. The full study, including the four phases, took place between March 2019 and March 2021.

### Original GMT Program

The original GMT program includes up to nine 2-h sessions, which include Presentation slides, therapist scripts, activity materials (e.g., worksheets and a deck of playing cards) and take-home workbooks. Sessions include character examples to demonstrate real-world concepts, in-session activities to promote experiential learning, and discussion of participants' own experiences. The take-home workbook includes “assignments”, such as monitoring for absentmindedness and related consequences (16).

The program developers report a seven-session version of GMT (16); however, the nine-session version of the program has been described elsewhere (28). Session 1 aims to help participants to be aware of absentminded errors in everyday life. Session 2 builds on absentminded errors and their associated consequences. Participants learn that they can avoid making slips by building their attention. Session 3 introduces the “automatic pilot”, an expression of habit that can be responsible for absentminded errors. In session 4, participants are taught to say STOP out loud to interrupt the automatic pilot and reduce slips. Session 5 introduces working memory as the mind's “mental blackboard”. Participants are taught to frequently check their “mental blackboard” to protect their goals from distraction. Mindfulness meditation is introduced to build awareness of feelings, behaviors, and goals. Session 6 builds on “STOP” and mindfulness and teaches participants to “state” their goals out loud. Session 7 introduces conflicting goals in the context of decision-making and encourages the use of ‘to-do” lists as a decision-making strategy. Session 8 introduces task-splitting to help participants to split unwieldy goals into more manageable steps. At this point, participants start to STOP-State-and then Split their goals. Session 9 encourages participants to Check their overall goals and to interrupt ongoing behavior that can interfere with goal achievement.

### Phase 1: Planning—Streamlining GMT

#### Participants

We recruited neuropsychology experts (*n* = 4) who were familiar with the original GMT program for a content re-development focus group. All participants were recruited from Monash University.

#### Materials

The core research team updated GMT modules and program contents to tap into the key cognitive deficits associated with substance use disorders, based on systematic reviews and meta-analyses (8, 15, 29).

**Figure 1:**
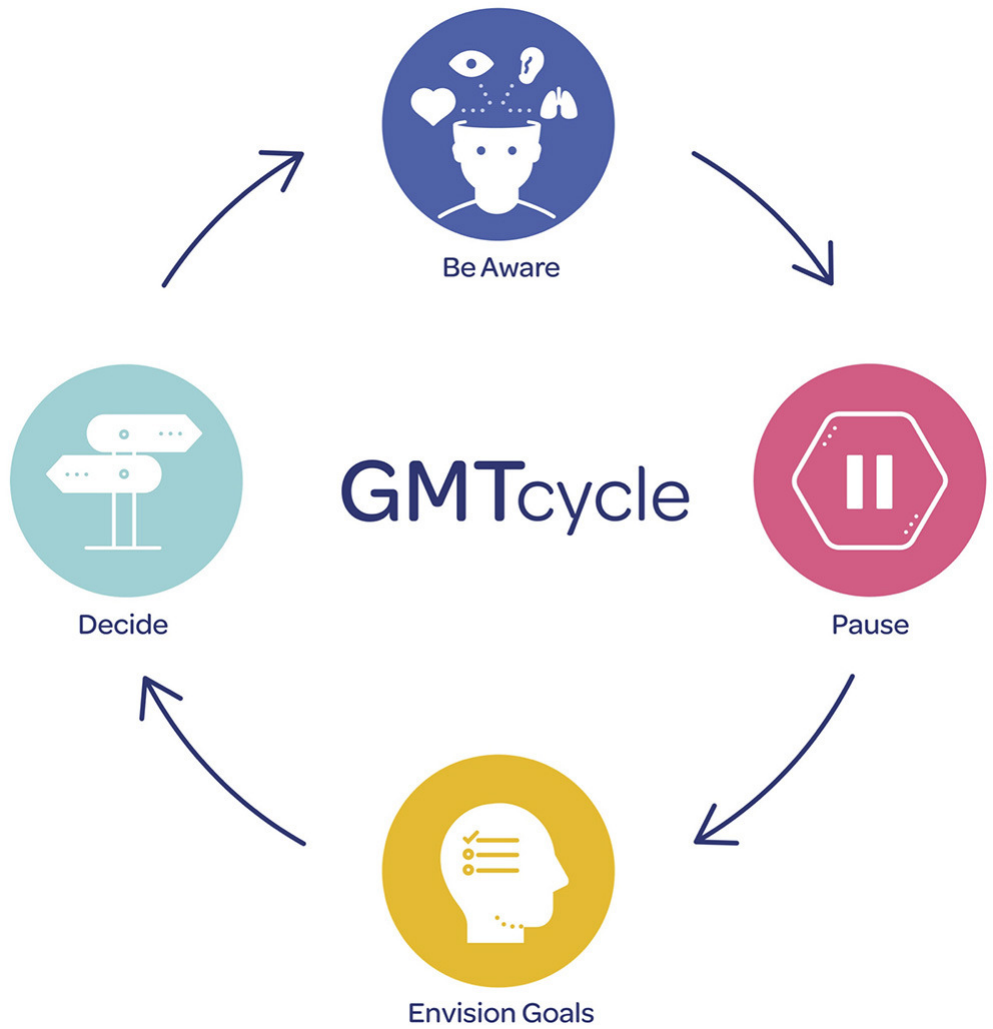
GMT^+^ cycle.

We restructured GMT into four 90-min weekly modules, each training a specific cognitive function. Module 1 (Be Aware) trains focused attention, module 2 (Pause) trains impulse control, module 3 (Envision Goals) trains goal setting, and module 4 (Decide) trains decision-making. See Supplementary Table 1 for a breakdown of the original (GMT) and updated (GMT^+^) content. GMT^+^ is designed as a 4-staged cycle (see Figure 1) that can be employed by consumers in any given moment. Participants can be aware of their attention and surroundings, pause and breathe, consider their goals (short-term or long-term) and make a decision.

The revised content includes a greater focus on building a longer-term mindset, hence strengthening the decision-making components of original GMT. An example of this was the incorporation of episodic future thinking (EFT) in module 4 (Decide). EFT involves imagining future events through guided instruction (30) and has shown efficacy at improving preference for larger delayed rewards over smaller immediate rewards in substance-using populations (31, 32).

We updated character narratives that were aimed to illustrate real-world concepts, to enhance the relevance for people with MUD (e.g., younger characters with relatable employment, interests, and relationship problems). We selected in-session cognitive activities from the original program that matched GMT^+^ training principles, such as a simple routine-based task that elicits errors due to inattention (Be Aware module), and a multitasking activity that highlights goal neglect due to distractions (Envision Goals module). We increased the rule complexity of these tasks to evoke the desired errors (“slips”) in people with MUD, who have milder deficits than people with brain injury. The newly developed activities in module 4 (Decide) were designed to help to participants to practice setting and staying on track with long-term goals. We also included new strategies to demonstrate learning (e.g., presenting problem scenarios and asking consumers to suggest the most appropriate GMT^+^ strategies).

#### Procedures

We conducted a 2-h face-to-face focus group with neuropsychology researchers and presented the updated modules and content via presentation slides. We sought feedback on the new program structure (i.e., streamlined contents and stronger focus on decision-making), whether the active GMT training ingredients were maintained and if the new elements were appropriate for the program, and whether the overall training ingredients were appropriate in the context of addiction. Facilitators (AA and AR) took written notes throughout the session to capture verbal feedback. After the focus group, the core research team (AVG, AA, AR) met to review the data that were collected.

### Phase 2: Intervention Design—Enhancing Engagement

#### Participants

We recruited consumers with MUD (*n* = 4; two women) to attend a face-to-face intervention design focus group. Participants were recruited from Turning Point, a public addiction treatment center based in Melbourne, or from eligible people who had previously participated in research with our group. Participants were compensated with a $20 grocery gift card. Eligibility criteria included a current or past diagnosis of MUD, aged 18 or over, and the absence of intellectual disability or severe neurological conditions. Seven participants consented to take part in the focus group, although three did not attend on the day.

#### Materials

We collaborated with design researchers in healthcare to reimagine the intervention materials and promote consumer engagement with the program. Design priorities were grouped into five categories, including material re-design, program delivery, enhancing program relevance, assessing acceptability of program delivery, and encouraging skills practice. We developed a range of fresh color palettes and fonts for the slide decks, new character designs, updated in-session cognitive task materials to encourage active engagement (e.g., sound buzzers, vintage cartoon cards), and a selection of written journal activities to gauge consumer preferences on reflective skills practice. We designed a GMT^+^ bracelet to serve as a visual reminder of the 4-staged cycle displayed in Figure 1, with the goal of encouraging participants to regularly practice GMT^+^ strategies to promote skill habituation.

#### Procedures

We conducted a 2-h focus group with consumers at Turning Point meeting rooms, employing think-aloud techniques. The format was a structured session with presentation slide content, guided questions, and ratings stickers to gauge preferences for select concepts. Focus group participants were asked to share ideas, interact with tasks and materials, and provide verbal feedback. The facilitators assessed participants' understanding by summarizing key points and checking for accuracy. Facilitators took written notes throughout the session to capture verbal and non-verbal feedback.

We sought feedback on material redesign (e.g., color palettes, fonts, logo, illustrations), ways to enhance program relevance (discussing personal goals and previous treatment experiences), delivery format (how comfortable participants were in contributing to the group), and enhanced skills practice materials (presenting new activities, assessing difficulty level and whether they produced desired errors). The core research team met after the focus group to review the data and implement the suggested changes for subsequent phases.

### Phase 3: Intervention Development—Prototype

#### Participants

Consumers with MUD (*n* = 5; two women) were recruited by contacting participants from the previous consumer focus group and through Turning Point for an online focus group. Three participants from focus group 2 (75%) returned for the follow-up session. Two additional participants were recruited “*de novo*”, to enable novel perspectives and prevent confirmation biases. Participants were compensated with a $25 grocery gift card for attending the focus group and an additional $15 grocery gift card for returning written materials. Eligibility criteria was the same as Phase 2. Participants required access to the Internet and a device to access the video conferencing software (e.g., smart phone, tablet, laptop). Six participants consented to take part in the focus group. On the day of the focus group, one participant did not attend.

#### Materials

**Figure 2:**
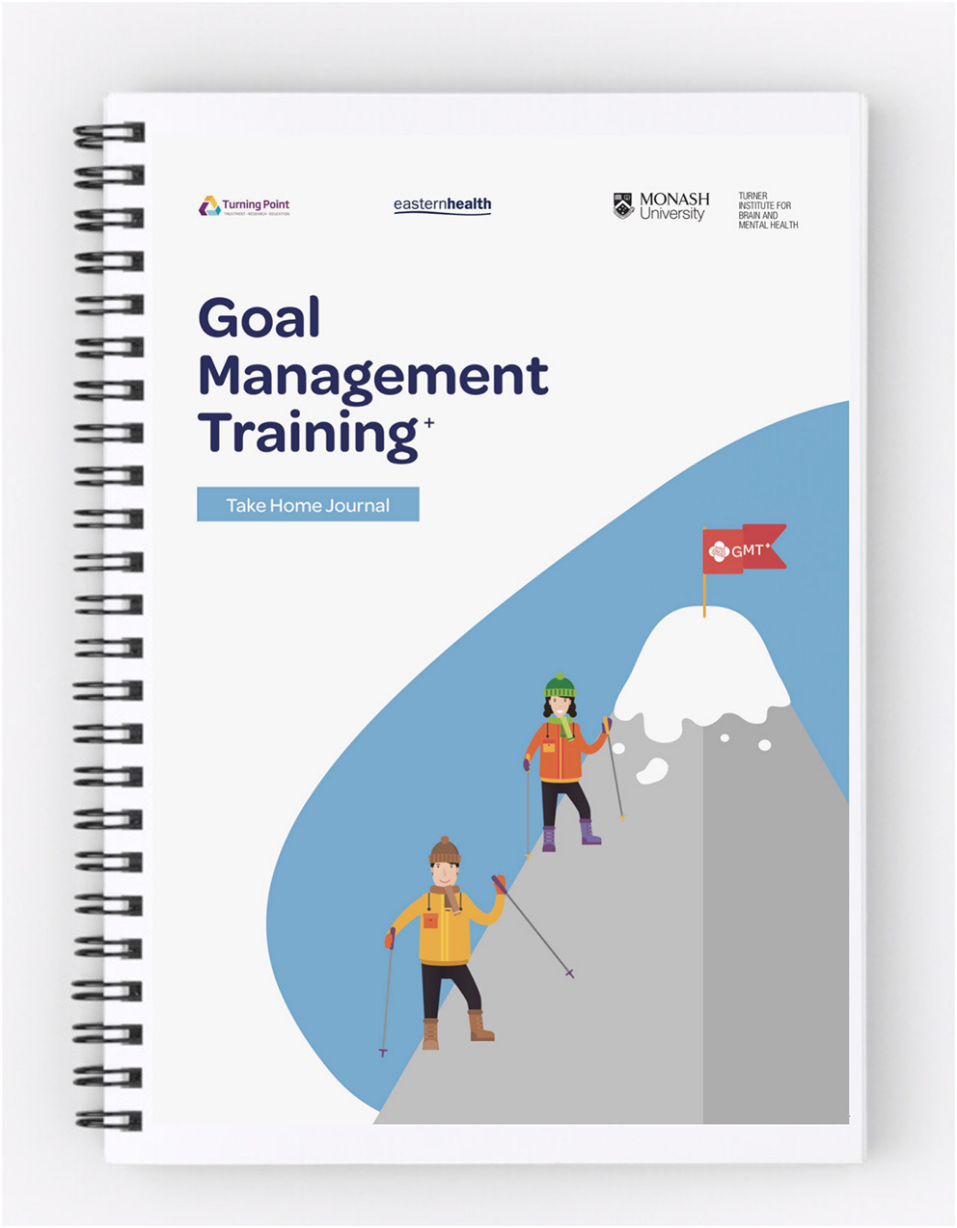
GMT^+^ journal.

We collaborated with design researchers in healthcare and developed a prototype of GMT^+^ to present in the intervention development focus group. The prototype included sample presentation slides, a new GMT^+^ ambassador character to demonstrate real-world progress throughout the program, and in-session activities relating to module 1 (Be Aware). We developed a printed journal with seven daily activities to encourage consumers to practice skills relating to module 1 (see Figure 2). This journal included both skills reflection activities (how participants used GMT^+^ skills in everyday life) and creative activities where they could practice GMT^+^ skills during the task (e.g., mindful drawing to regulate breathing).

#### Procedures

The focus group duration was 2 h and was conducted online, using videoconferencing software. The structured session included presentation slide content, guided questions, and ratings polls to gauge preferences. The prototype of module 1 was embedded in the session. We tested acceptability of the program and materials, the delivery format (including feasibility of an online delivery format), engagement with concepts and activities, engagement with the between-session journal, and the appropriateness of language. Qualitative aspects included observation of consumer engagement and their interaction style, and reviewing content themes that arose from think aloud techniques. Quantitative aspects included Likert scales to indicate acceptability of the journal content. The session was audio recorded and facilitators took written notes to capture non-verbal feedback.

Following the focus group session, we mailed out packs to attendees with the sample GMT^+^ journal and a pre-paid return envelope. Participants were asked to complete the journal daily, which was designed to take ~1 h in total. A summary of the key training concepts was included in this journal. Participants were asked to time themselves completing each activity, to rate their engagement (Likert scales), and to provide written feedback. After completing the journal, participants were asked to mail it back to the researchers. Two participants returned the completed journal including their feedback.

### Phase 4: Intervention Development—Clinical Acceptability

#### Participants

Clinicians (*n* = 2) were recruited from Turning Point and The Turner Clinics, Monash University for an online program review and feedback session. We invited clinical directors of these treatment services, due to their high level of knowledge around the needs of people with MUD and the implementation of new interventions. Neither treatment service was involved in the future pilot trial.

We also engaged clinicians (*n* = 9) from three treatment services (including metropolitan and regional locations) that had agreed to take part in the future trial to refine aspects of the final intervention delivery.”

#### Materials

Clinical psychology researchers and design researchers in healthcare collaborated to develop the complete package of program materials, including presentation slides and presenter scripts, in-session activities, and journal activities for the four modules. We developed journal activities that appeared “enjoyable” to complete, whilst training the relevant cognitive skills. The journal activities for each week targeted building attention and meta-cognition (week 1: Be Aware), learning to pause and gain control over impulsivity (week 2: Pause), improving focus on current and short-term goals (week 3: Envision Goals), and improving future-focused reflection and long-term decision-making skills (week 4: Decide).

#### Procedures

We conducted a 2-h online videoconference review session with clinical treatment providers to assess reactions to the program and to further optimize acceptability and feasibility. We presented the full program including: the in-session presentation slide material and activities, and the between-session journal activities for each of the four modules. Facilitators paused for discussions during the presentation to collect ongoing feedback from clinicians. The session was audio and video recorded. Following the review session, the core research team met to discuss the clinical feedback and to incorporate the final changes to the program materials.

We then conducted meetings with clinicians from treatment centers involved in the trial to present the final program and seek feedback on intervention delivery. Following these meetings, the core research team met to discuss the feedback and to incorporate minor changes to the presenter scripts and facilitator roles during the group sessions.

## Results

### Phase 1: Planning—Streamlining GMT

#### Focus Group 1: Neuropsychology Researchers

Neuropsychology experts were positive about the revised 4-session program structure and design, including GMT^+^ characters, who faced similar challenges to those typically encountered during MUD recovery. The experts further helped to select key learning activities (e.g., promote participant involvement in helping GMT^+^ characters to solve a problem) and approaches to delivering the content (e.g., including a mixture of theory, discussions, and practical activities). Key feedback from the session (see Supplementary Table 2) highlighted the importance of helping participants to habitualize the training concepts and to guide participants around how specific strategies (e.g., Pause) could be used in “hot” (i.e., in high level of emotionality) contexts. We responded to this feedback by prioritizing the between-session journal engagement as a tool for skill habituation and agreed on the need to develop a visual reminder to employ strategies in everyday situations.

### Phase 2: Intervention Design—Enhancing Engagement

#### Focus Group 2: Consumers With MUD (Face to Face Session)

**Figure 3:**
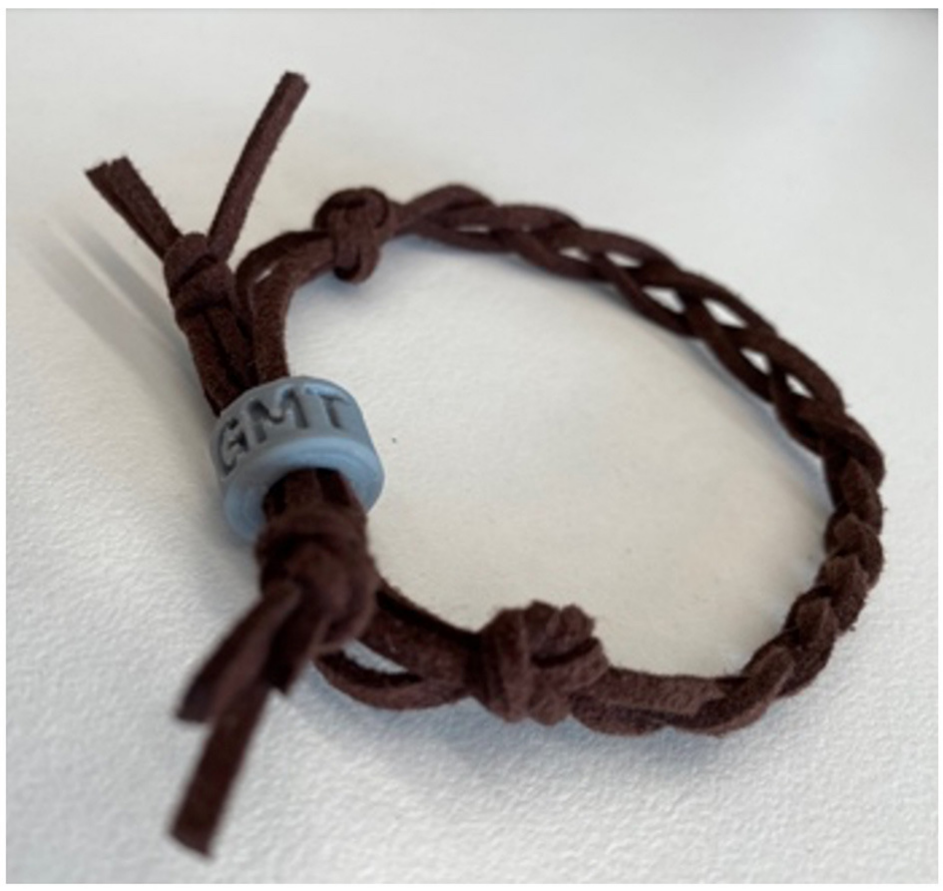
GMT^+^ mindfulness bracelet.

**Figure 4:**
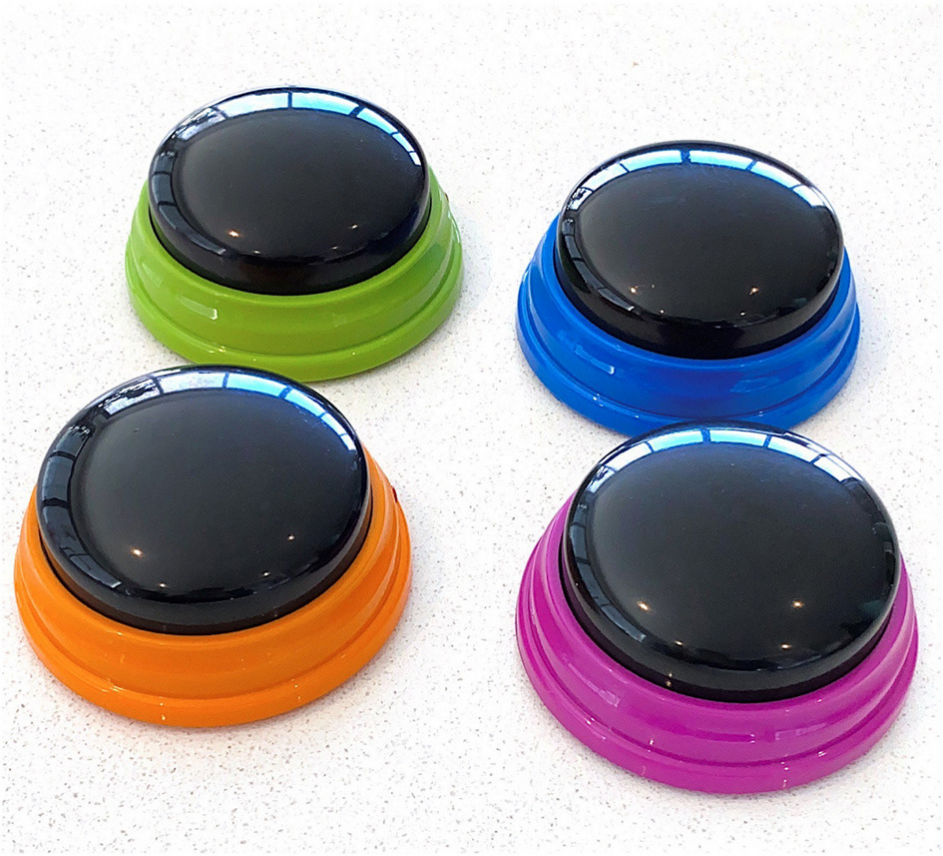
Interactive hand buzzers replaced a “hand-clapping” task to enhance engagement.

Participants with MUD endorsed the novel four-staged cycle of Be Aware-Pause-Envision Goals-Decide and considered GMT^+^ to be a valuable type of intervention that is currently missing from addiction treatment services. The results indicated initial acceptability of the journal activities, the GMT^+^ bracelet (see Figure 3), and the group-based format that included discussions with and interaction between group members and facilitators. Interaction with the revised cognitive activities (see Figure 4 for an example) indicated that they appropriately elicited the desired errors to demonstrate executive dysfunction (e.g., missing specific details on the cartoon sorting cards). Participants thought the activities were aligned with the desired purpose and were enjoyable, with a minimum average enjoyment rating of 7/10 for each one.

Qualitative themes and program changes are summarized in Supplementary Table 3. Key feedback included examples of relevant personal goals and the need for simplified journal activities. We used this information to facilitate relevant goal-related discussions and developed journal activities that permitted consumers to focus on working toward multiple goal categories throughout the program (e.g., short term and long term, or across different areas of life). We also prioritized the development of single-focused creative activities that appeared relevant.

### Phase 3: Intervention Development—Prototype

#### Focus Group 3: Consumers With MUD (Teleconferencing Session)

**Figure 5:**
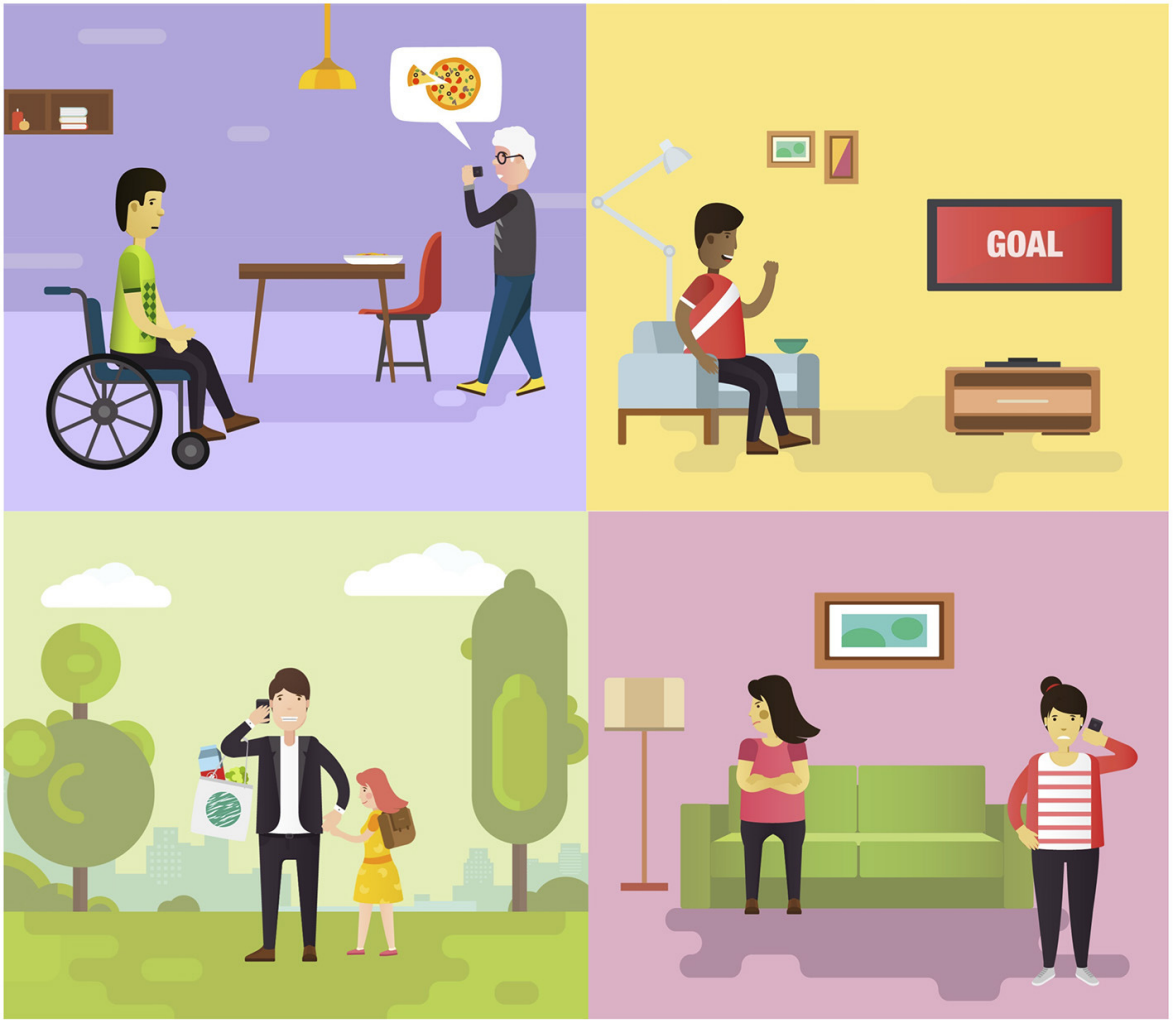
Examples of diverse GMT^+^ characters.

Participants with MUD who had attended focus group 2 were positive about how we had incorporated their previous feedback and expressed enthusiasm for the redesigned materials. Sharing how participants' feedback was implemented is important for building trust in and establishing commitment to the participatory design approach (26). Feedback from focus group 3 (see Supplementary Table 4) indicated overall acceptability of the language used, the interactive group intervention format, and the content that was tailored to the needs of people with MUD. However, a key theme that emerged from the think aloud strategy was the need to further enhance the relevance of the program to people who are undergoing treatment for MUD. This included a greater focus on group discussions and characters with more relevant problems for this population. We responded to this feedback by addressing substance use more frequently in planned group discussions, developing a range of GMT^+^ characters with different demographics, attributes, and goals (see Figure 5), and inviting consumers to select their preferred GMT^+^ ambassador character to relate to in-session.

Participants indicated acceptability of the journal and affirmed that they would complete it if enrolled in the GMT^+^ program. Quantitative feedback indicated that the activities were enjoyable (mean rating 7.2/10), helped participants to be more aware of their attention (mean rating 6.9/10), and most activities helped them to focus on their personal goals (mean rating 6.5/10). Both participants indicated that they felt confident completing the journal activities based on the in-session strategies, discussions, and instructions provided. Finally, participants felt that the types of activities and the language used were appropriate. Participants did not complete the journal daily and one participant stated that they would be more likely to complete it regularly in an inpatient treatment setting. Completion rates and feedback highlighted the importance of reviewing journal activities at the beginning and end of each session to enhance motivation and to discuss individual experiences.

**Figure 6:**
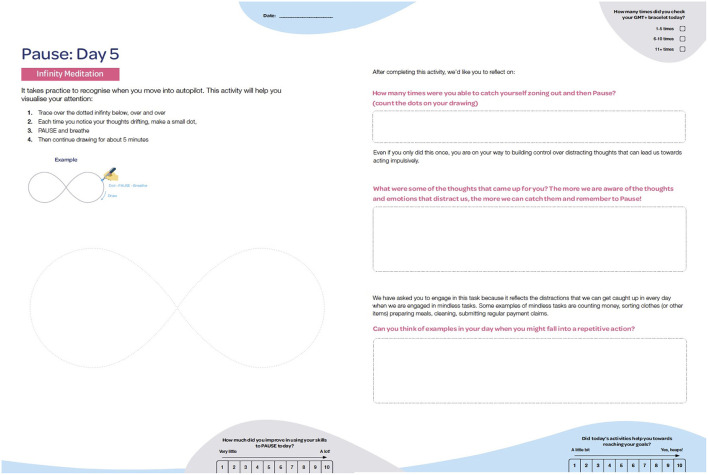
A creative journal activity and guided questions to connect the relevance to everyday life.

We also received important feedback on ways to improve the journal. Qualitative responses indicated that although the updated activities were creative and engaging (e.g., drawing a repetitive pattern to invoke autopilot), they required greater explanation about the relevance to GMT^+^ skills and everyday life. Participants enjoyed reflecting on their journal work and often provided written content beyond the provided space, suggesting a need for more reflection space on relevant pages. We addressed this feedback by providing a debrief page after each activity that explained its relevance and allowed consumers to reflect on how it may relate to their own life (see Figure 6). We increased the reflection text entry space across all activities.

### Phase 4: Intervention Development—Clinical Acceptability

#### Review Session With Clinicians

Clinical treatment providers in the final review session were positive about the need for this type of intervention within addiction treatment services and considered the intervention feasible to implement. Specifically, one clinician commented favorably about how theory has been interwoven into practice “to make the whole package usable for the end-consumer.” The activities were also considered “fresh” and engaging, and appropriate for addiction treatment as the content of the stimuli is far-removed from drug-related stimuli (e.g., replacing playing cards with cartoon and travel post cards). Session feedback is outlined in Supplementary Table 5.

Key feedback included the need to normalize errors and slips to consumers who may be particularly sensitive to making mistakes and develop related feelings of hopelessness. We addressed this feedback by highlighting how these errors are experienced by all people and provided relatable examples of mistakes by facilitators. Clinicians also highlighted that one activity which promoted multi-tasking (even if tasks were not completed) may foster complacency with unachieved goals and reinforce procrastination. We included a debrief script to reinforce the objective of making progress toward multiple goals at the same time (to avoid neglecting one goal in favor of another).

Clinicians from the three participating treatment centers were positive about the final program materials and content, how we had addressed prior clinician feedback, and the feasibility of implementing the 4-week program within their treatment service. Key feedback included the benefit of discussing addictive behavior more broadly during group discussions, due to the high prevalence of comorbid substance use disorders, and to assess participant comprehension regularly due to low literacy levels in some clients. We modified the presenter scripts and group discussion points to enable a discussion of different examples of habitual behavior during active addiction. This included examples relating to methamphetamine use and other substance or behavioral addictions. We also divided roles of the facilitators during written tasks and when introducing journal activities, to allow one facilitator to present the content and a second facilitator to provide individual support to group members.

### Key Intervention Modifications

We have made a number of changes to GMT^+^ throughout the planning, intervention design and development phases. Some of the major changes included: (1) developing more relevant and meaningful program characters and narratives to help demonstrate everyday problems where GMT^+^ may help; (2) adapting the language to more appropriately connect the concepts to everyday life for people with MUD; (3) developing more engaging and cognitively appropriate in-session activities; (4) developing a completely new between-session journal to increase the “hands-on” enjoyment of regular skills practice; and (5) including new strategies to develop a future focused mindset to help with decision making. We describe the final GMT^+^ intervention, guided by Items 3–10 from the TIDieR checklist (see Table 1).

**Table 1:** Description of in-session content, in-session activities and between-session journal activities for the four GMT^+^ modules.

**Module**	**In-session content**	**In-session activities**	**Journal activities**
Be aware	Introduction to GMT^+^. Participants select their preferred GMT^+^ ambassador character (3 choices). This character demonstrates errors and progress throughout the GMT^+^ program. Defining simple goals and complex goals. Defining short-term goals and long-term goals. GMT^+^ ambassador character: Introduce a situation where the character was not focussed on their overall goal and became distracted and made “slips”. The group discusses potential consequences and similar personal experiences Introduce the concepts of “zoning out” and “autopilot”; how they differ; and how they can lead to slips. Practicing present mindedness. Five-minute breath meditation script. Discuss how present mindedness can prevent “zoning out” and “autopilot” Introduce this week's journal activities.	Bouncer buzzer: participants are instructed that they are a bouncer at a venue and have been tasked to press their buzzer to let every name into the venue, except “David”. Names appear in succession. Task is designed to foster mistakes by pressing the buzzer for David. Mindfulness bracelet: participants weave their own GMT^+^ bracelet; mindfully being aware of this creative experience. They then wear the bracelet on their wrist as a reminder to practice GMT^+^ skills. Activity is designed as a visual leave behind reminder to encourage habitual use of GMT^+^ skills.	Signature on repeat: Participants detect when they slip into autopilot when writing their signature repetitively. They then draw their attention to deliberate changes to their signature. Zentangles: Participants draw repetitive shapes and notice when they have zoned out by detecting changes in the shape. Goal bubbles: Participants write their small and big goals in different areas of life (e.g., family, career, hobbies). Cone of awareness: Participants practice present-moment awareness and reflect on their thoughts while sitting silently for 5 min. Time to Reflect: Participants reflect on how they used different GMT^+^ skills that day (complete 3 × per week).
Pause	Recap previous material and discuss journal completion. Demonstrate how “slips” occur in a card sorting activity. Introduce “Pause” as a way to interrupt “zoning out” or being in “autopilot” mode. Discuss making a habit out of Pause. Participants are prompted to use their GMT^+^ bracelet as a reminder to Pause. Linking the breath to Pause (Pause then breathe), practicing breath meditation. Discuss how Pause could have helped the GMT^+^ ambassador character in week 1. Link Pause to emotionally aroused moments (“hot contexts”). Discuss character example of acting without thinking when emotionally aroused. Group discusses potential consequences and shares similar personal experiences. Discuss examples of everyday situations when it is important to Pause and breathe (e.g., when stressed or tired). Introduce this week's journal activities.	Cartoon card sorting: participants quickly divide cards into two piles according to a specific rule. This task demonstrates how slips can happen in routine tasks. The task is then repeated with a reminder to Pause to help reduce the number of slips made. Bouncer Buzzer: repeat bouncer task from Be Aware module, with occasional reminders to Pause to reduce the number of slips made. Facilitators highlight client progress from last week.	Draw your breath: Participants draw a continuous line to reflect the in-breath (draw upwards) and out-breath (draw downwards). This continues across the page, with the goal that the line becomes smoother, reflecting slower and more relaxed breaths. Body maps: Participants draw where they feel stress, anxiety, anger, or excitement in the body. These are cues of the autopilot taking over. Infinity meditation: Participants draw a repetitive shape and Pause, drawing a small mark, each time they have zoned out or entered autopilot. Dot-to-Dot (picture of a tiger): Participants are instructed to breathe and Pause after every five dots to maintain focus and avoid making an error in the drawing. Time to Reflect: Participants reflect on how they used different GMT^+^ skills that day (complete 3x per week).
Envision Goals	Recap previous material and discuss journal completion. Introduce “mental notes”, which are the goals in our working memory. These mental notes (goals) are fragile messages in our mind that can be overwritten by distractions. Participants learn to check their “mental notepad” to protect their main goal. Character examples of how distractions can cause us to neglect our goal if we're not actively focusing on it. Highlights practical (late for work) and emotional (strained relationships) consequences. Participants are taught how to become aware of distractions and to refocus on the main goal in their mental notepads. Envisioning short-term goals: Stating goals out loud and visualizing goals as words or pictures in the “mental notepad”. Participants visualize achieving short-term goals ahead of time. Introduce this week's journal activities.	Vintage travel card sorting: Participants sort the cards into two piles according to a specific rule. Distractor words are called out during the task and participants must note when the same word is called twice. The task is then repeated with a reminder to Pause to help reduce the number of slips made. Multitasking activity. A series of activities are introduced and participants are given a short time limit to complete the task. The main goal is to attempt each task and bonus points are provided if this goal is achieved. The task is designed to distract from the main goal as each task is detailed and time-consuming to complete. The multitasking activity is then repeated with the main goal explicitly highlighted as the key focus. Participants state the goal out loud before starting and occasionally throughout the task. Improved performance on the task is highlighted to the group.	Goal distractors: Participants consider their biggest goal distractors and ways to overcome these distractions (using GMT^+^ skills). Short-term goal: Participants write one goal for the next day and then visualize achieving it. They later reflect on whether this goal was achieved. Word search: Participants complete a word search and focus only on the goal words, ignoring various distractor words. Returning to goals: Participants practice returning to a specific goal multiple times throughout a task. This task is designed to encourage participants to frequently return to other goals during the day. Blackout poetry: Participants are tasked to black out all words in a short passage except five words that will form a short poem. Task is designed to train focussed attention and working memory. Time to Reflect: Participants reflect on how they used different GMT^+^ skills that day (complete 3 × per week).
Decide	Recap previous material and discuss journal completion. Introduce decision-making and long-term goals. Long-term goals can be interrupted when (1) there is uncertainty around what to do, or (2) when the decision is clear, but motivation is low. Using SMART goals to plan for long-term goals. Task-splitting long-term goals into manageable steps. Managing multiple long-term goals. Participants are prompted to consider their goal priorities and to create a hierarchy of most important to least important goals. Introduce Episodic Future Thinking (EFT) to visualize achieving long-term goals in order to aid short-term motivation. Recap of entire program: Participants are presented with different scenarios where characters have implemented some GMT^+^ strategies but have forgotten at least one important component. Participants discuss which GMT^+^ strategies could help the character. GMT^+^ ambassador character: Participants return to the GMT^+^ character they selected and offer suggestions to prevent the initial slips this character faced in session 1. Summarize key content of GMT^+^ and introduce this week's journal activities.	SMART goals: Participants create SMART goals for a goal that they would like to achieve in the next 6 months. The goal should be Specific, Measurable, Achievable, Relevant, and Time specific. Activity is designed to help participants with long-term goals when they are unsure about where to start. Daily planner: Participants create a daily planner to manage multiple long-term goals. Designed to avoid goal-neglect when there are multiple important long-term goals. Episodic Future Thinking exercise. Participants envision a future experience of achieving a goal in 3 months. Guided future meditation exercise to evoke rich detail about the experience of achieving a future goal.	SMART goals: Participants create a SMART goal for a goal that they would like to achieve in 1 month. Goal distractors: Participants consider distractors that may interfere with achieving long-term goals when they feel triggered toward using substances. They reflect on different GMT^+^ skills that can help to manage distractors. Relationship goals: Participants creatively reflect on a future relationship goal by drawing a picture. Visualize: Episodic Future Thinking activity to reflect on detailed aspects of achieving a future goal. Designed to help participants to pre-experience this feeling of goal achievement. Time to Reflect: Participants reflect on how they used different GMT^+^ skills that day (complete 3 × per week).

### Goal Management Training^+^

The final version of Goal Management Training^+^ (GMT^+^) is a manualized therapist-facilitated targeted cognitive remediation group program. We developed a final kit comprising a range of materials to administer GMT^+^. The in-session materials include presentation slide content with manualized presenter scripts for each of the four modules, guided discussions, audio recordings of meditation scripts, and activity materials (e.g., sound buzzers, sorting cards). The between-session material includes a printed spiral-bound journal to distribute to each participant, consisting of daily activities that relate to the weekly module (i.e., Be Aware, Pause, Envision Goals, Decide) for them to complete. Participants record their responses in the journal. Session 1 (Be Aware) demonstrates how errors (“slips”) can occur during moments of inattention. Participants learn the difference between “zoning out” (daydreaming or not paying attention) and “autopilot” (acting in a state of habit) and are taught to use mindfulness strategies to bring their attention back to the present when they begin to zone out. They make a GMT^+^ bracelet to serve as a visual reminder to employ these skills. Session 2 (Pause) teaches participants to regularly “breathe and Pause” to prevent zoning out or acting on autopilot. Participants consider the benefits of using Pause in emotional contexts to avoid negative consequences of habit-driven behavior. Session 3 (Envision Goals) introduces the “mental notepad” (a concept to represent working memory), where fragile goals are stored. Participants are taught to state and visualize their main goals to protect them from distractions. Session 4 (Decide) introduces short-term and long-term decision-making, and common barriers to implementing decisions. Participants are taught to set achievable goals, to break goals into manageable steps and to vividly pre-experience the achievement of a salient future goal to aid motivation. Table 1 outlines a breakdown of content, in-session activities, and between-session journal activities. GMT^+^ is a variation of copyrighted material. Provided that the original developers agree, materials can be requested for research purposes by contacting the corresponding author.

GMT^+^ facilitators should undertake training and be familiar with the content and manuals prior to administering the intervention. GMT^+^ is designed as an in-person (face-to-face) intervention that should be delivered on-site by two facilitators at an inpatient addiction treatment facility. The facilitators will need access to a projector screen and computer (to run the presentation slide content), tables (as participants work with physical materials) and a quiet room. There are four weekly sessions that run for 90 min and should be delivered in groups of 4–8 program participants. A 15-min break is provided half-way through the session to minimize fatigue. The journal contains detailed instructions for daily completion and does not require any input or participant monitoring from staff at the treatment facility. Between-session journal completion is discussed as a group at the beginning of sessions 2–4 and the facilitator will assess for daily completion.

## Discussion

This study aimed to update GMT (a cognitive remediation program for brain injury) to tailor it to the key cognitive deficits and treatment context of MUD, and to maximize users' engagement with its contents and delivery. We utilized an evidence- and person-based approach, collaborating with neuropsychology experts, designers in healthcare, people with MUD and clinical providers to develop the novel GMT^+^ program. Results from our four-phased approach provide initial evidence that GMT^+^ may be engaging for consumers and may be feasibly applied in addiction treatment settings. These findings illustrate the benefits of the evidence- and person-based approach and provide greater confidence to move into the evaluation phase with a protocol that has considered potential risks, for example, barriers to implementation, or a lack of consumer engagement.

The initial focus on four cognitive processes (i.e., attention, impulse control, goal setting, decision-making) was endorsed by neuropsychology experts and consumers. We selected these four components based on consistent evidence revealing impairments in these specific executive functions, as well as their relationship with key addiction treatment outcomes (8, 9, 11, 33). One of the primary goals of addiction treatment is to help individuals to develop self-control strategies to manage cravings and emotionally salient situations, skills that inherently rely on these prioritized cognitive processes.

The updated GMT^+^ program trains these components through strategy learning, fostering skill practice in everyday situations, and reinforcing a simplified reflection-action cycle to employ in any given moment [Be Aware (of inattention)- Pause (to prevent acting on autopilot)- Envision Goals (to prevent goal distractions)- Decide (to consider longer-term outcomes)]. Consumers build on these skills as they progress through the program, providing an opportunity for skill acquisition and mastery through self-initiated practice and active reflection regarding action selection and analysis of potential related consequences. Unlike some existing interventions that train specific cognitive processes through repetitive task practice, e.g., computerized working memory or inhibitory control training (34, 35), GMT^+^ teaches consumers to group multiple skills together and apply them in ecologically relevant situations (for example, noticing drug-cravings, taking a deep breath, and bringing attention back toward long-term goals).

Neuropsychology experts highlighted the need to help participants to habitualize strategies to promote their effectiveness in critical real-world situations. We addressed this suggestion by including a GMT^+^ mindfulness bracelet to serve as a specific visual cue to promote a stimulus-response association (36), for example, noticing the bracelet and applying “Pause”. As this bracelet is always accessible to consumers, it may enhance the chance of successful habit formation while participants are in the early phase of skill acquisition (37). We also incorporated activities in the take-home journal to encourage consistency of practice and skill mastery (37, 38). For example, a checkbox to note how many times consumers used their bracelet each day, activities to reflect on how different GMT^+^ skills were employed that day, and creative activities that were superficially enjoyable (e.g., dot-to-dot, mindful drawing) but required the use of GMT^+^ skills to complete.

Consumers provided input on their personal goals during recovery and emphasized the need to develop relatable characters and scenarios. We used this feedback to create a range of character profiles and situations where a character may have benefited from applying GMT^+^ strategies. We provided consumers with the opportunity to personalize their group treatment by selecting their preferred GMT^+^ ambassador character to enhance meaning and motivation to engage in group discussions (39). Consumers indicated a preference for simple and creative journal activities with a clear purpose. We responded to this feedback by creating intuitive and enjoyable activities requiring minimal instructions and including clear links to everyday challenges for consumers to reflect on. We reason that these activities will be well-suited to promote strategy habituation due to anticipated adherence to regular journal completion (skills practice). Amending the language and visual representation of key strategies was an important aspect of our development process. For example, consumers preferenced the word “Pause” over the original “Stop,” which was considered punitive, for labeling Module 2.

The final showcase to clinicians identified important unintended effects, for example, GMT^+^ uses multitasking (even if all activities are not completed) and task errors as ways to illustrate challenges for executive function; however, these aspects may trigger procrastination and perceived hopelessness. We addressed this feedback by enhancing the context around these activities (i.e., explaining the aim as “making progress toward goals”). Facilitators are also prompted to share personal accounts of everyday “slips” to group members and to normalize mistakes on the tasks as part of the learning process, with the goal of reducing feelings of shame or a tendency to overidentify with making errors. An additional benefit might also be to promote self-compassion during a fragile treatment period where self-criticism may be more likely to lead to earlier program drop-outs and re-engagement in addictive behaviors (40, 41).

### Strengths and Limitations

There were several strengths to our design process. We employed four diverse groups in the development process, including neuropsychology experts, design researchers in healthcare, consumers with MUD, and clinical service providers. We conducted both face-to-face and online focus groups with consumers, providing an indication of positive engagement with future online administration. Our person-based, participatory design perspective valued and incorporated the needs and experiences of the end-consumer at each intervention development phase. In addition, our intervention development model has synergies with Stage 0 (the evidence-based research guiding our content changes) and Stage I (intervention refinement, involving the target population and clinical providers, and preparation for pilot testing) of the established NIH Stage Model for Behavioral Intervention Development (42). This provides confidence that we have followed the recommended initial steps in intervention development and are now ready to move into pilot testing.

There were some limitations to our work. There were only four participants in focus group 2 and five participants in focus group 3, potentially limiting the perspectives provided from consumers with MUD. We included participants with both current and past methamphetamine use. It is possible that the perspectives of those who no longer used methamphetamine are not representative of a population currently undergoing treatment. However, this concern is balanced by the benefit of receiving perspectives from people who understand the treatment process and have maintained abstinence. There were also only two clinicians in our intervention review session, which limits clinical perspectives on the finished program. However, clinicians from the addiction treatment services involved in the pilot trial agreed with the feedback and subsequent changes and endorsed the final intervention materials. Further, we only tested a prototype of module 1 (Be Aware) due to time constraints. Although we tested key concepts from other modules (e.g., Pause, the mental notepad, EFT), the presentation slide materials and journal activities for modules 2, 3, and 4 were not delivered in their final form to the end-consumer. However, clinical service providers were positive about the final materials for all four modules.

### Future Research

The main components of GMT^+^ are relevant not only to MUD but also other substance use and addictive disorders. This research may inform the development of modified interventions for different addictions, incorporating similar evidence- and person-based design principles. This paper also highlights the importance of collaborating with end-consumers prior to administering existing evidence-based interventions in different consumer populations. We employed several changes to design, training concepts, relevance of characters, and how the strategies were practiced between sessions to aid habituation.

There is also a potential to expand this intervention to treat other mental health needs where there are executive dysfunctions and difficulties with goal-related decisions, for example, binge eating disorder, ADHD, or schizophrenia (14, 43, 44). This program may also be appropriate to apply to other populations where shorter administration times and less content repetition are indicated (e.g., OCD) (28). To adapt the intervention for these groups, content changes would need to apply, such as tailoring character examples to these groups and raising discussions about how GMT^+^ skills could be applied for everyday difficulties. Consultation with specific service providers and relevant consumer groups could assist with these changes.

We now plan to commence a proof-of-concept pilot trial to determine the feasibility and acceptability of GMT^+^ as an adjunct inpatient addiction treatment for MUD. This trial will indicate the benefit of GMT^+^, compared to psychoeducation-control, at improving executive functions and clinical outcomes including treatment retention, substance use, craving, and quality of life (specific details are included in the trial registry; Trial ID: ACTRN12621000172808).

## Conclusions

We have presented the systematic development of GMT^+^, an updated version of GMT that is tailored to key cognitive deficits and treatment requirements for MUD. GMT^+^ is a 4-week group program with a between-session journal to foster everyday skill practice. It includes clear and practical strategies to employ in everyday situations and is designed to improve attention, impulse control, goal setting and decision-making in MUD. By employing an evidence- and person-based approach, we have demonstrated how potential barriers to engagement and uptake by consumers can be addressed through modifications to the intervention content, materials, and delivery format. As such, we are confident that we have developed an intervention with initial acceptability for the treatment of MUD. However, further research is now required to further assess acceptability and feasibility, and the efficacy of GMT^+^ in a pilot trial.

## Data Availability Statement

The raw de-identified data supporting the conclusions of this article will be made available by the authors, without undue reservation.

## Ethics Statement

The studies involving human participants were reviewed and approved by Monash University Human Research Ethics Committee and Eastern Health Human Research Ethics Committee. The patients/participants provided their written informed consent to participate in this study.

## Author Contributions

AA: conceptualization, data curation, investigation, methodology, project administration, visualization, writing—original draft, and writing—review and editing. AR: conceptualization, data curation, project administration, investigation, and writing—review and editing. EP and BK: data curation, project administration, investigation, resources, and writing—review and editing. DF: project administration, resources, and writing—review and editing. DL: funding acquisition, supervision, and writing—review and editing. AV-G: conceptualization, data curation, investigation, methodology, funding acquisition, supervision, and writing—review and editing. All authors contributed to the article and approved the submitted version.

## Funding

This study was funded by Monash Addiction Research Centre and the National Centre for Clinical Research on Emerging Drugs Research Seed Funding Grant (NCR3SF10). AA and AR are funded by the Australian Government Research Training Program. DL was supported by an NHMRC Investigator grant (1196892). AV-G was supported by a Medical Research Future Fund, Next Generation of Clinical Researchers CDF2 Fellowship (MRF1141214).

## Conflict of Interest

AV-G has received funding from Servier for consultancy work and Elsevier for editorial work. No pharmaceutical grants were received in the development of this study. DL has provided consultancy advice to Lundbeck and Indivior, and has received travel support and speaker honoraria from Camurus, Indivior, Janssen, Lundbeck, Shire, and Servier. These organizations do not stand to benefit from this project. DL has been an investigator on an untied education grant from Sequirus, as well as an investigator-led grant from Camurus, both unrelated to the current work. The remaining authors declare that the research was conducted in the absence of any commercial or financial relationships that could be construed as a potential conflict of interest.

## Publisher's Note

All claims expressed in this article are solely those of the authors and do not necessarily represent those of their affiliated organizations, or those of the publisher, the editors and the reviewers. Any product that may be evaluated in this article, or claim that may be made by its manufacturer, is not guaranteed or endorsed by the publisher.

## Abbreviations

MUD: Methamphetamine Use Disorder
GMT: Goal Management Training
GMT^+^: Goal Management Training^+^.
