# Supplementary material for: Development of Goal Management Training+ for Methamphetamine Use Disorder Through Collaborative Design
Source: Front Psychiatry. 2022 Apr 25;13:876018. doi: 10.3389/fpsyt.2022.876018 (PMC9082590; doi:10.3389/fpsyt.2022.876018)
Supplement: Supplementary file 1 [file Data_Sheet_1.PDF]

Supplementary Material

Supplementary Table 1. ***Revised program structure for GMT+***

GMT		GMT+	
**Module**	**Objectives**	**Module**	**Objectives**
1. The Absent/Present Mind	Overall introduction.<br><br>Defining simple & complex goals.<br><br>Absentmindedness and slips.<br><br>Participants are introduced to a recurring GMT character, an absentminded older professional.<br><br>Goals of GMT and introduction to training workbook.	1. Be Aware	Overall introduction.<br><br>Defining simple & complex goals.<br><br>Participants are introduced to a recurring GMT+ "ambassador" character who has received treatment for MUD.<br><br>Zoning out and autopilot – how they lead to slips.<br><br>Present-mindedness (focusing on the breath) to interrupt the autopilot.<br><br>Goals of GMT+ and introducing the daily journal.
2. Absentminded Slip-Ups	The consequences of making slips.<br><br>Why we make slip-ups.<br><br>Building attention to avoid making slip ups.<br><br>Learning to monitor attention and slips in different situations.	2. Pause	Why we make slips<br><br>Learning to "Pause" and breathe when we start to zone out or are acting on autopilot<br><br>Strategies to practice "Pause".<br><br>Mindfulness meditation practice.

			Using Pause in emotional situations.<br><br>Learning to make "Pause" a habit to prevent slips or negative consequences.
3. The Automatic Pilot	Defining the automatic pilot.<br><br>Autopilot errors – errors that occur when we are acting out of habit.<br><br>Stopping the autopilot by being presentminded.<br><br>Introduction to the breath and body scan to create distance from the automatic pilot.	3. Envision Goals	Mental notes are the goals in our working memory.<br><br>These mental notes (goals) are like fragile messages that can be overwritten by distractions.<br><br>Being aware of distractions and refocusing on the main goal can prevent mental notes (goals) from fading.<br><br>Envisioning short-term goals: stating and visualizing goals as a message, and visualizing achieving goals ahead of time.
4. Stop the Automatic Pilot	Introduction to stopping the automatic pilot.<br><br>Making a habit of stopping the automatic pilot by saying STOP out loud.<br><br>Practicing focusing on the breath to improve present-mindedness.	4. Decide	Using long-term goals to guide current decisions to competing goals.<br><br>Breaking goals down by creating SMART goals and task-splitting goals.<br><br>Managing multiple goals and competing goals.<br><br>Envision your future goal – applying episodic future thinking to stay motivated in progressing towards a future goal.
5. The Mental Blackboard	Working memory and the mental blackboard.<br><br>Distractions cause our goal (or task instructions) to roll off the blackboard.		

	Using STOP to check the mental blackboard before it rolls up and the instruction disappears.
	Building attention awareness (STOP) and attention control (check).
6. State Your Goal	Getting side-tracked or distracted from your main goal.
	Refreshing the mental blackboard by stating goals out loud.
	Introduction to "STOP" (present-mindedness)-STATE cycle.
7. Making Decisions	Examples of conflicting goals.
	Emotional reactions to competing goals, including indecision.
	To-Do Lists in the "STOP"-STATE cycle.
	Strategies for preventing indecision.
8. Splitting Tasks into Subtasks	Emotional reactions to complex tasks.
	Splitting up complex tasks into smaller steps.
	"STOP"-STATE-SPLIT cycle.
	Prioritizing subtasks.
9. Checking ("STOP!")	Examples of tasks that didn't go to plan.
	Importance of checking the overall goal.

	"STOP"-STATE-SPLIT cycle (with Check).<br><br>Learning to adapt to changes in circumstances (external or internal).

Supplementary Table 2. ***Key feedback from the neuropsychology focus group and subsequent changes to the intervention planning.***

Theme	Feedback	Program modifications
**New program content (abbreviated format and greater decision-making focus)**		
Integrating theory with practice	Link the theory back to everyday behaviors that lead to drug-taking problems.	Included summary of the program's success in previous trials with addiction to promote buy-in at the start of session 1.
	Minimise theory delivered in-session and maximise on ways of practicing GMT+ strategies.	Provided top-level theoretical information and then raised discussions about how this may relate to the group members.
	Participants will need buy-in to the program, rather than too much theory. Explain the benefits at the beginning of the first session and consider talking about success stories.	Increased examples of how and when GMT+ skills can be practiced in everyday life.
Future goals need to be achievable	It is important to focus on achievable goals for participants. Reduce the possibility that they might set themselves up for failure with unachievable goals.	Included exercises to help participants to think about goals that are achievable within 1 day, 1 month, 3 months, and 6 months.
		Increased in-session facilitator involvement to help participants to set realistic goals.
Anchor Pause to the Breath	Anchor the Pause command to relaxation and mindfulness.	Increased repetition of Pause-Breathe as a training concept in-session.
	One way to help generalise the skills to everyday life is to anchor them to the breath.	Prioritised focusing on 'mindfulness' in the design phase to encourage regular focus on the breath.
**Appropriateness of new training ingredients in the context of GMT**		
Testing new tasks and task variations	Experts suggested testing the task revisions to see whether they are too complex for people with MUD.	Placed a priority on consumer testing of cognitive activities in the design phase.
		Assess whether they foster desired errors, or if they are perceived as too difficult.<br>-

Focus on task-splitting for decision-making	Task-splitting [from the original program] is important, particularly when participants are faced with rewarding decisions.	Increased focus on task-splitting complex goals in module 4 (Decide).
**Relevance for addiction**		
Reduce focus on past experiences	Avoid suggesting that participants look to the past to be reinforced by past behaviors and avoid talking about the past too much in group discussions [due to negative past experiences].<br><br>People with substance use disorders who have negative experiences may be highly sensitive to what hasn't worked.	Reduced decision-making based on past consequences.<br><br>Increased focus on future-based guided imagery experiences to visualise achieving a long-term goal (future-based motivation).
Enhancing relevance of language	Suggestion to update the language of training concepts.<br><br>For example, using everyday phrases like 'drifting thoughts', rather than 'absentmindedness'.	Compiled different names for GMT concepts to test in the design phase.<br><br>Language was placed as a priority for assessing acceptability.
The importance of Pause for MUD	This was considered as the most important concept by experts.<br><br>It will be important to help participants to habitualise this concept.<br><br>Regular attendance and skills practice will be key to skill habituation.	Provide in-session examples of when to "Pause" in everyday life.<br><br>Encouraging attendance and between-session skills practice through engaging design.
Pause is important in the context of emotional valence	The program needs to help participants to focus on stopping thoughts about highly rewarding actions.	Increased in-session content about Pausing when feeling triggered or stressed.<br><br>Placed a priority on designing engaging skills practice activities to reinforce Pausing when feeling emotionally aroused.

Supplementary Table 3. ***Key feedback from the design focus group with people with MUD and subsequent changes to the intervention design.***

Theme	Feedback	Program modifications
**New material design**		
Bold/contrasting colours	Preference for strong, bold, juxtaposition of colours.	Selected a colour palette with contrasting shades for each module.
	Group preference for the colour pink.	Included pink as one of the core colours in the cycle.
	The selected contrasting colours were considered to reflect participant goals (contrasting to reflect committing to the goal or not committing to the goal).	Developed a new logo based on selected colours.
Diversity of character illustrations and positive emotions in characters	If people can't relate to the illustrations, they will switch off and disengage from the program.	Included dogs in the illustrations.
	Some participants identified with including children in stories, others did not. Participants indicated varied interests (e.g., sports, driving, families).	Increased diversity of characters (different skin colours, abilities, ages, family structures).
	All participants expressed a preference for including dogs/pets.	Reflected different interests/hobbies in illustrations.
**Enhancing program relevance**		
Varying goals	Goals varied between participants, from finding employment to giving up smoking. Goals were personalised and extended beyond not using methamphetamine.	Included in-session discussion of varying types of goals participants might have.
		Activities to focus on different goal categories (e.g., health, hobbies) and different sized goals (small and big).
Future goals/dreams	Thinking and dreaming about future goals was valued.	Included a 'future me' creative writing/drawing section at the beginning of the journal.
	Preference for reflecting on goals after achieving addiction-related goals, for example goals reflecting the next phase such as career and family.	Included an activity to draw your future relationship goals in the journal.
**Enhancing skills practice**		

Reduce creative journal task complexity	Collective feedback that complex tasks [e.g., multi-step task connecting colours, emotions, and films] were frustrating/confusing.	Replacing complex tasks featuring multiple steps with activities with a single focus (one step).
	Uncertainty around the purpose of complex tasks and how they connected back to the program.	Enhanced focus on explaining the purpose of each task.
Leave-behind mindfulness bracelet	Unanimous preference for the mindfulness bracelet. All participants stated that they "loved this activity".	Addition of this new activity into the final program.
	Preference for using leather material for the bracelet.	Increased the amount of time creating the bracelet in-session, playing relaxing music (mindful cues), and increased focus on explaining the benefit and importance of wearing the bracelet every day.
	Reflection that this activity was a personal experience and communicated a message to treat themselves well (labelled a 'recovery bracelet').	
Disappointment around making errors on the in-session tasks	Reflection that making a mistake on cognitive tasks resulted in focusing on failures and reduced attention.	Increased the amount of time devoted to debriefing the task and explaining that they are designed to be difficult and promote errors.
	Consensus that the tasks were useful to demonstrate making mistakes.	Increased focus on improved performance on the task after applying GMT+ strategies.
New activities considered engaging	The new buzzers were considered fun.	We modified existing cognitive activities with new materials.
	The vintage cartoon cards were favoured and were considered to provide additional nostalgic value.	Replaced 'clapping hands' with fast sound buzzers in the final program. These are used to demonstrate 'slips' when we zone out.
	Participants were slow to sort through the cartoon cards as they enjoyed looking at these cards.	Replaced a deck of cards with cartoon post cards (card sorting activity 1) and vintage travel post cards (card sorting activity 2).
		Included a time limit on the card-sorting activity to encourage fast sorting of engaging material.

Striking the right balance with activity complexity	Participants provided feedback on the sorting rules, and all agreed that a geography sorting rule on a vintage postcard sorting task was too difficult.<br><br>They preferenced sorting by letters, colours, or other visual details instead.	Participants tasked to sort cards into two piles according to a specific letter rule, e.g., place all cards with ‘m’ in one pile, all other cards in the other pile.

Table 4. ***Key feedback from the development (prototype) focus group with people with MUD and subsequent changes to the intervention.***

Theme	Feedback	Program modifications
**Acceptability of content**		
Increasing methamphetamine use content	Group consensus to increase the focus on substance use and how GMT+ can be used to prevent relapse following treatment.	Increased information in the program introduction about how GMT+ relates to treatment goals following MUD.
	It is important to acknowledge addiction throughout the program and help people to build goals to move past their addictions. Don't ignore 'the elephant in the room'.	Increased daily examples of how to use GMT+ when facing challenges related to MUD (cravings, lapses).
		Included content and activities about how GMT+ can help during the first week out of rehab.
GMT+ ambassador character needs to be relatable to each group	Feedback that the ambassador character is too middle class and is not relatable enough. Group members shared that some people may be homeless before going to residential rehabilitation.	Developed three diverse characters to represent the GMT+ program. A summary of these character is presented to each group at the start of session one and group members can choose the most relevant character that will be carried through the remaining program material for their group.
	As the character example is placed at the beginning, group members suggested that the first hurdle they face could be overcoming drug use and to then set up goals to help the character to move on from there.	Included relationship difficulties in one of the character examples.
		Included a discussion about why the group chose that character and whether they could relate to the situation.
	Relationship changes are a large part of methamphetamine use. Getting back in touch with family and social connections is a common goal. Group preference to increase focus on relationship goals from the beginning of the program.	
	Increasing opportunities for people to talk about their own experiences more may increase engagement during a period where capacity to concentrate is limited.	
Increasing the connection between creative activities in the journal and everyday life	Feedback that it was difficult to understand the purpose of some creative activities.	Included information at the beginning of the journal to explain how to fill it in.
		Linked tasks to everyday life, e.g.:

	Suggestion to include more information to explain creative journal activities.	*'We have asked you to engage in this task because it reflects the distractions that we can get caught up in every day when we are engaged in mindless tasks. Some examples of mindless tasks are counting money, sorting clothes, preparing meals, cleaning, and submitting regular payment claims. Can you think of examples in your day when you might fall into a repetitive action?'.*
Simplify the 'working memory' concept	Group preference for the concept 'mental notepad' to represent working memory.<br><br>Mental notepad was considered relatable to all people and could be applied to their lives.<br><br>Group members related the concept to writing notes, shopping lists, or daily plans.<br><br>Group discussion around the idea that the mental notepad was not permanent (i.e., the messages can fade due to disruptions). Group members were positive about the idea of mentally "rewriting" the message on the mental notepad to keep messages in mind and to continue working on goals.<br><br>An alternative computer image was considered to look like hard work to use as an ongoing concept.<br><br>Preference for consistency between the terminology and visual representation, e.g., if using 'mental notepad' as a term, the visual should also reflect a notepad filling up with messages.	Selected 'mental notepad' to replace the mental blackboard from original GMT for module 3 (Envision Goals).<br><br>Visualised working memory as a thought bubble in the mind that fills up with thoughts, goals and distractions. Consumers are instructed to make the goal stronger by going back and visualizing their mental notepads.
Episodic Future Thinking could be further enhanced	The group response to this activity was very positive.<br><br>Some participants found it difficult to think of an initial goal before completing the task.	Increased the amount of time setting up the EFT activity in-session (module 4) and explaining the purpose – to develop a future mindset.<br><br>Increased the number of scripted prompts during the guided visualisation.

	Participants suggested sharing goals with other group members afterwards to enhance the meaning of the activity. This was also considered to be an opportunity to help foster personal relationships within the group setting.	Inserted a discussion following the activity to share goals and individual experiences while engaging in the EFT activity.
	The guided prompts were considered helpful to bring more visual detail into the future memory (for example, prompts on where the future goal is happening or who is there when the goal is achieved).	
	The activity was considered a positive ‘reality check’ for whether current lifestyle choices are in line with their long-term goal.	
**Acceptability of delivery format**		
Online delivery of GMT^{+} using a videoconferencing platform will require the opportunity to connect with other group members	Preference for the face-to-face group setting from focus group 2 over the online setting as it permitted greater opportunity for group members to get to know each other and to share stories and goals (fostering connection).	Included instructions in the program guidelines for any online variations to include videoconferencing ‘breakout rooms’ – these are sessions that are split off from the main videoconference meeting, allowing smaller groups to meet.
	One suggestion for improving the online delivery was to include smaller breakout rooms to facilitate connections between group members. This was considered a helpful solution by the group.	Increased the number of in-session group discussions and interactive activities where participants worked in pairs.
	Suggestion to increase general discussions and the opportunity to engage with people in an online format so that people felt like they were being listened to.	
**Language**		
Language: Pause, not Stop	General preference for ‘Pause’ rather than ‘Stop’.	Replaced ‘Stop’ from the original program with ‘Pause’.
	‘Pause’ was considered an invitation to be gentle to yourself.	Included an in-session group discussion around other personally meaningful words to remind participants to pause and take a breath.

	‘Stop’ was considered as too harsh and group members felt like it communicated that they were doing something wrong.<br><br>The group liked the idea of linking ‘Pause’ with the breath (taking a breather and then continuing on).	
‘Zoning out’ is relatable	‘Zoning out’ was selected by most group members to communicate the concept of being absentminded.<br><br>Group members all agreed that this concept was very important in addiction as people can feel triggered and make rash decisions when they are zoning out (for example, ringing their dealer without awareness).	The concept of being absentminded is described as ‘zoning out’ in the final intervention.<br><br>Increased focus on why this is important in the context of addiction recovery in the in-session content.<br><br>Included a discussion about any other words that mean the same concept to participants in order to increase personal relevance.

Supplementary Table 5. ***Key feedback from clinical service providers and subsequent changes to the final intervention.***

Theme	Feedback	Program modification
Normalizing errors on the cognitive tasks	People with addiction are hard on themselves when they make a mistake.	Increased focus on normalizing errors and communicating that many people regularly make these types of slips.
	It is important to normalise that all people make these mistakes.	Explained why it is important to focus on in the context of addiction recovery but used modified language to soften the message.
Important to help participants manage multiple long-term goals	People in addiction treatment can become overly focussed on one or two of the goals and end up neglecting the other goals.	Introduced a brief activity in module 4 (Decide) to task-split multiple goals so that participants can work towards achieving small steps within multiple goals each day.
	Consider how you can help participants achieve one of their goals without neglecting the other goals.	
Multitasking activity may send a confusing message as the goal is to attempt all activities, even if they are not completed within the allocated time	People with MUD may often start multiple tasks without finishing them and may often move between tasks. They may feel as though they're failing because they never complete anything.	Provided clearer instructions prior to commencing the task that the purpose is to practice multitasking.
	The key here is to be clear about what the task is about – multitasking.	Debrief afterwards to explain that the goal was not to leave each task incomplete, but to focus on the overall goal when working with multiple tasks or components at once.
